# The making of evidence-informed health policy in Cambodia: knowledge, institutions and processes

**DOI:** 10.1136/bmjgh-2017-000652

**Published:** 2018-06-22

**Authors:** Marco Liverani, Kannarath Chheng, Justin Parkhurst

**Affiliations:** 1 Department of Global Health and Development, London School of Hygiene and Tropical Medicine, London, UK; 2 National Institute of Public Health, Phnom Penh, Cambodia; 3 London School of Economics and Political Science, London, UK

**Keywords:** health policy, health systems, qualitative study

## Abstract

**Introduction:**

In global health discussions, there have been widespread calls for health policy and programme implementation to be informed by the best available evidence. However, recommendations in the literature on knowledge translation are often decontextualised, with little attention to the local systems of institutions, structures and practices which can direct the production of evidence and shape whether or how it informs health decisions. This article explores these issues in the country setting of Cambodia, where the Ministry of Health has explicitly championed the language of evidence-based approaches to policy and planning.

**Methods:**

Research for this paper combined multiple sources and material, including in-depth interviews with key informants in Phnom Penh and the analysis of documentary material and publications. Data collection and analysis focused on two key domains in evidence advisory systems: domestic capacities to generate health policy-relevant evidence and institutional mechanisms to monitor, evaluate and incorporate evidence in the policy process.

**Results:**

We identified a number of structural arrangements that may increasingly work to facilitate the supply of health-related data and information, and their use to inform policy and planning. However, other trends and features appear to be more problematic, including gaps between research and public health priorities in the country, the fragmented nature of research activities and information systems, the lack of a national policy to support and guide the production and use of evidence for health policy, and challenges to the use of evidence for intersectoral policy-making.

**Conclusions:**

In Cambodia, as in other low/middle-income countries, continued investments to increase the supply and quality of health data and information are needed, but greater attention should be paid to the enabling institutional environment to ensure relevance of health research products and effective knowledge management.

**Key questions**

**What is already known?**

- There is widespread recognition that policy and planning in the health sector should be informed by the best available evidence; however, our understanding of the institutional and structural arrangements that can promote improved knowledge utilisation is limited, especially in low/middle-income countries (LMICs).

**What are the new findings?**

- Our study takes an institutional approach to examine the local systems of structures and practices influencing the production and utilisation of health evidence within the country setting of Cambodia.
- We found that increasing availability of health data and research products, combined with recent reforms of the health system, may increasingly serve an evidence advisory role in the health sector.
- However, lack of clear guidelines and weak domestic research capacities make the use of evidence in line with best practices less likely.

**What do the new findings imply?**

- In Cambodia, as in other LMICs, continued investments to increase the supply and quality of health policy-relevant data and information are needed, but more attention should be paid to the underlying structural arrangements including the strengthening of local research organisations as well as the development of policy and local institutions that can facilitate knowledge management and translation.

## Introduction

In current global health policy discussions, there has been increasing emphasis on the importance of promoting the use of health data and research findings to inform policy formulation and implementation. Particular concerns have been expressed about the need for a "culture of evidence" in low/middle-income countries (LMICs), where "the pressure to extract the most out of funds is particularly great, as the gap between the resources available and those that are needed to address the burden of preventable diseases is larger than elsewhere" (Rosenbaum *et al*, p54).^1^ In such contexts, a systematic approach to policy and planning, informed by a rigorous and transparent evaluation of relevant data, information and knowledge, is thought to be crucial at different stages in the policy process, from the identification of public health priorities to the development of cost-effective, equitable and sustainable solutions to address them.^2^ In LMICs, however, domestic capacities to enable evidence-informed approaches tend to be less well established than in high-income countries,^3^ including the capacities to generate and use evidence and the capacities to routinely produce high-quality data on population health and the health sector that are needed to make reliable and meaningful evidence claims.^4^

In recognition of these gaps, several initiatives have been developed in LMICs to improve health information systems, facilitate interactions between producers and users of health data and research, and thus encourage a more systematic approach to policy-making. Such efforts have often focused on improving the capacity of individual groups—in particular, researchers and/or decision-makers—and their ability to generate, transfer or receive research evidence.^5–7^ In addition, the problem of getting research into policy has been the focus of many case studies of knowledge translation—also known as knowledge transfer, knowledge management or knowledge exchange.^8 9^ Systematic reviews of these works have synthesised lessons to increase the likelihood of uptake of pieces of research in health (or other social) policy-making.^10^

A clear insight emerging from the literature is that strategic interventions to improve evidence uptake may risk a lack of impact if the underlying institutional and structural arrangements are not well understood.^11 12^ Yet, findings and recommendations in the literature on knowledge translation are often decontextualised and tend to prioritise strategies for researchers to have better links with decision-makers, for decision-makers to better understand research or for efforts to be made to bridge the gap between these groups. Comparatively much less attention has been paid to the nature of evidence and when it meets public health priorities in the countries and to the local systems of structures and rules which can direct how evidence is used. A different approach to evidence use has alternatively been promoted by Dobrow *et al* who reflect on how various contextual factors shape the introduction, interpretation or application of evidence.^13^ Yet despite awareness of these issues, two recent reviews have found gaps in the literature, with few empirical works studying the use of health evidence within actual political contexts and few studies explicitly considering institutional factors shaping evidence use in health policy-making.^14 15^

This article aims to contribute to a better understanding of these issues within the country setting of Cambodia. Over the past two decades, sustained efforts have been made in this country to reform health policy and the organisation of the health system in order to address health challenges and the provision of equitable health services. These efforts, combined with the effects of steady economic growth, have contributed to a general improvement in population health, particularly in the areas of infectious diseases, child and maternal health; however, key challenges remain, including gaps in the public health infrastructure, the increasing burden from non-communicable diseases and injuries and the threat of antimicrobial resistance.^16^ In the process of health reform, the Ministry of Health (MoH) has explicitly highlighted the importance of evidence-based policy-making to identify and meet strategic objectives. In particular, the second Health Strategic Plan (2008–2015), which defines priorities and goals for the entire health sector, highlighted the need "to strengthen and invest in health information system and health research for evidence-based policymaking, planning, monitoring performance and evaluation" (p13).^17^ And yet, Cambodia faces a number of challenges in establishing and implementing a system through which relevant evidence can inform health policy decisions.^18^ Despite high rates of economic growth, Cambodia is still a fairly low-income country (only recently rising in its World Bank classification from low to lower-middle income) with limited bureaucratic capacity and infrastructure. International organisations such as donors or global health bodies may have particular influence and control over the use of evidence in this environment, or Cambodian political priorities and needs are often set centrally without necessarily reflecting the goals of evidence-based health policy that have been championed by the MoH and the global community alike.^19^ Further, despite the use of a common language of evidence-based policy in national documents, there are, in fact, many types of evidence which can speak to different political concerns, while different pieces of evidence and different constructions become prominent and translated into knowledge for action, given the politics involved in policy decisions, the institutional context of decision-making and the system of knowledge production.^20^

In consideration of this, the present report specifically examines challenges to, and opportunities for, the promotion of evidence-informed approaches to health policy-making in Cambodia, with particular attention to the structures, mechanisms and contextual factors that shape the production and utilisation of evidence. After a description of concepts and methods, the following sections report findings from interviews with key informants and the analysis of associated documents. In the discussion, we then reflect on the implications of our research for institutional development in the country and the wider context of global health policy.

## Methods

This paper is part of a larger evaluation of evidence utilisation for health policy in Cambodia, which included a system-wide exploration of institutional and contextual issues (reported here) and the analysis of three specific case studies of evidence use (reported in a previously published paper).^20^ Research for this paper was informed by a conceptual framework including two key domains. First, we considered local sources of evidence that are used to inform knowledge claims about health issues and policy options to address them. Specifically, we considered the health information system, since this is an essential source of evidence for decision-making and the allocation of scarce resources optimally^21^; we also focused on domestic research capacities, given their recognised importance for the generation of evidence which responds to health priorities and needs in the countries.^22^ Second, we considered institutional arrangements which may affect when, how or in which ways those pieces of evidence can inform decisions—particularly in terms of institutional bodies, the links between them and their rules or mechanisms of functioning. In researching these domains, attention was paid to the historical background, in keeping with a growing body of empirical works which have documented the important effects of the past on the subsequent development of domestic institutions, reform and capacities, in public health as in other sectors.^23 24^

Data collection and analysis were informed by the principles of exploratory case study research, an approach commonly used in policy studies which combines multiple sources and material to gain a better understanding of a particular issue within a given context, and generate insights and concepts for further inquiry.^25^ Specifically, we conducted in-depth interviews with policy-makers in the MoH and other key informants with extensive knowledge of the health sector in Cambodia, who could provide an overview of systems and structures in place to generate and use evidence as well as expert views about achievements, challenges and opportunities. Following interviews with a set of initial informants, identified for their central role in health policy development, additional participants were recruited by snowball sampling or purposively selected to explore emerging issues further. In total, we approached by email or phone contact 21 potential participants, but 5 did not respond to our request for an interview. Interviewees included health sector managers in central departments of the MoH or other institutional structures (n=5), managers of local research organisations (n=3), representatives of international organisations based in Cambodia (n=5), consultants (n=2) and 1 director of a local non-governmental organisation (NGO). Interview schedules were flexible and lightly structured around the following themes, depending on the role and expertise of each individual informant: (1) the nature and source of evidence in the country, including routine data collections and health research; (2) institutional mechanisms and processes for decision-making in the health sector and the way in which evidence is presented and evaluated in these processes; (3) views on challenges to and opportunities for strengthening the evidence advisory system in Cambodia. All interviews were conducted face-to-face by ML (alone or together with KC and/or JP) in Phnom Penh between April and September 2014. Informed consent was obtained for every participant. Where additional consent was given, interviews were taped and then transcribed; otherwise, extensive notes were taken during and after the meeting. Transcripts were coded using QSR NVivo 10 software and structured within the key domains of our investigation described above; open coding was also used to enable a broader reading of data and the identification of emerging issues within the given domains through an inductive, iterative approach.^26^ In addition to the interviews, documentary material and publications were reviewed at different stages to gain a better understanding of the research themes, relevant historical developments and factual information to clarify particular points. Documents were identified based on the extensive experience of two authors in the country, with additional searches in PubMed by themes related to the issues under investigation or sourced from key informants at the MoH or international organisations. Reviewed documents included policy papers, health sector reviews, published and unpublished reports and academic articles, and institutional websites. Preliminary findings were presented at the international symposium "Building research capacity for Cambodia", held in Phnom Penh in September 2015, where feedback was received. In the presentation of findings below, structured around the key domains in our conceptual framework, anonymised citations are included to illustrate key points and referenced by the unique identifier CAM-*n, date*.

## Results

### Health data, research and state reconstruction

#### Health information system

In the early 1990s, Cambodia embarked in a process of political reform and state reconstruction following two decades of civil conflict. In 1993, the first general elections, overseen by the United Nations Transitional Authority in Cambodia, laid the foundation for democratic transition and the peaceful resolution of conflicts. After the elections, the new government drafted the Constitution of the Kingdom of Cambodia, which established a constitutional monarchy, based on a bicameral legislative system and multiparty elections.^27^

Cambodia’s political transition ended a long period of isolation, opening the country to greater engagement with the international community and the inflow of foreign aid assistance. Subsequently, significant investments have been made to rebuild and reform institutions for national planning and state administration, including structures for the collection and management of basic demographic data.^28^ The central office for statistical work, which was discontinued during the Khmer Rouge regime (1975–1979), was reorganised as a national institute in the 1990s under the Ministry of Planning, leading to the first national census (1998) after a gap of 36 years. From the 1990s, the National Institute of Statistics has issued other reports on demographic trends such as the Cambodia Socio-Economic Survey.

In the health sector, the development of a national information system was a priority in the reconstruction agenda. A pilot system for the collection of basic data on illness and service utilisation from public health facilities was established in 1993 with the support of the WHO and the Unicef.^29^ Subsequently, the National Health Information System (NHIS) was expanded and then the infrastructure has undergone several improvements, including integration of different reporting methods into a single format, computerisation at provincial and district level and standardisation in line with WHO guidelines.

The MoH has recognised the importance of strengthening the NHIS as a necessary requirement to support evidence-based policy-making, identifying gaps and challenges that should be addressed to improve the reliability and policy relevance of the system. As reported in the second national health sector strategic plan, however, the lack of comprehensive information technology (IT) coverage, human resources and capacities remains a national challenge.^17^ Today, data are still collected using paper registries in many health facilities, especially at the community level. As a result, the use of such data for statistical analysis requires a laborious process of data entry in electronic databases, which is prone to incompleteness. One informant further explained that large volumes of paper records that were collected in hospitals before the introduction of IT systems are not organised for research purposes, preventing the analysis of historical trends on the burden and characteristics of disease (CAM-03, 17 June 2014).

The NHIS is also unable to capture data from the private sector (eg, private clinics, pharmacies), which altogether account for 67.1% of first treatments in Cambodia.^30^ However, the implementation of periodic Demographic and Health Surveys (in 2000, 2005, 2010 and 2014) has contributed to a more accurate mapping of the health status and health-seeking behaviour of the Cambodian population. One informant noted that “the DHS is the most important piece of evidence for health policy in Cambodia,” explaining that the publication of findings showing high rates of maternal mortality in the 2005 DHS was crucial to generate policy attention to this problem (CAM-12, 25 August 2014). Surveillance and epidemiological data are also routinely collected, processed and published by the Communicable Disease Control department and specialised centres under the MoH. However, we found that the surveillance infrastructure is fragmented, with parallel data collection systems even for the same disease and lack of integration between them. A list of institutional data collection and reporting systems is provided in table 1.

**Table 1:** Institutional health data sources in Cambodia

Data source	Institutional body	Notes	Availability and/or reporting
National Health Information System	Department of Planning and Health Information, Ministry of Health http://www.hiscambodia.org	Routine data collection from public health facilities on illness and health service utilisation.	Annual report on Health Statistics Cambodia.
Cambodia Demographic and Health Survey (DHS)	National Institute of Statistics (Ministry of Planning); Directorate General for Health, Ministry of Health http://www.nis.gov.kh	Nationally representative household survey on key demographic and health indicators, including morbidity and mortality, healthcare-seeking behaviour, health expenditures, gender issues and disease awareness.	2000, 2005, 2010, 2014.
Cambodia Early Warning and Response System (CAMEWARN)	Department of Communicable Disease Control, Ministry of Health www.cdcmoh.gov.kh/	National surveillance system for 10 diseases, based on weekly reports from health centres, referral hospitals and two paediatric hospitals.	Weekly reports.
Malaria Information System	National Center for Parasitology, Entomology, and Malaria Control (CNM), Ministry of Health http://www.cnm.gov.kh/	Data on malaria diagnosis and treatment from Village Malaria Workers. Routine reports also include data from the National Health Information System.	Monthly bulletin, quarterly and annual reports.
HIV/AIDS Monitoring System	National Center for HIV/AIDS, Dermatology, and Sexually Transmitted Infections (NCHADS), Ministry of Health http://www.nchads.org/	Routine data collection on HIV/AIDS and STI prevention, care, support and treatment from all treatment centres as well as counselling and prevention sites.	Quarterly and annual reports.
TB Reporting System	National Center for Tuberculosis and Leprosy Control (CENAT), Ministry of Health http://www.cenat.gov.kh	Routine data collection from public health facilities for both tuberculosis and multidrug resistant tuberculosis.	Quarterly and annual reports.
National Census	National Institute of Statistics, Ministry of Planning http://www.nis.gov.kh	Micro datasets can be accessed for research purposes at the online repository system of the National Institute of Statistics (http://nada-nis.com) after authorisation.	By law, the general population census in Cambodia must be conducted every 10 years (1998, 2008). An intercensal population survey was conducted in 2004.
Cambodia Socio-Economic Survey (CSES)	National Institute of Statistics, Ministry of Planning http://www.nis.gov.kh	Key survey on living conditions in Cambodia. Results from CSES are used for monitoring the National Strategic Development Plan. The 2004, 2009 and 2014 surveys were based on large samples (about 12 000 households).	The CSES was conducted intermittently in the period 1993 to 2004 but since 2007 the survey is annual.

STI, sexually transmitted infection; TB, tuberculosis.

#### Research capacities

The development and current status of domestic research capacities in Cambodia has also been shaped by pivotal events in its modern political history. The establishment of national universities was initiated after independence by Prince Sihanouk, who founded the Royal Medical School (1953) and the Royal Khmer University (1960). As part of the project of nation building and modernisation, efforts were subsequently made to promote the professionalisation of medical research in the country such as the foundation of the *Société Royale de Médicine du Cambodge*, which published scholarly proceedings in its periodical bulletin.^31^ During the 1970s, however, academic institutions were banned under the Khmer Rouge regime, resulting in the disappearance of an emerging national research community. In recent years, resources have been allocated to reactivate and strengthen the educational curricula of national universities. In addition, local institutes and organisations have increased their research activities with the support of international grants and projects, including the University of Health Sciences, the National Institute of Public Health and the Cambodian Development Resource Institute, forming a new generation of qualified Cambodian researchers. Yet, research remains marginal in the national agenda and budget.^32^ As a result, local research institutions find it difficult to attract and retain skilled researchers. One local informant explained:

It is difficult to attract good researchers, as they prefer to work in the private sector or move abroad. (…) Staff can top up their salaries with grant money, up to USD 1000 a month. Still, this is not competitive enough to attract good researchers, because the market standard is around USD 3,000 (CAM-03, 17 June 2014).

From the early 1990s, a diverse range of international actors has filled this gap in domestic research capacity, generating research products on different aspects of health and healthcare in Cambodia. International actors that have financed or conducted health research include NGOs, academic institutes, private foundations, aid agencies, UN agencies and other international bodies such as the European Commission, research institutes or companies and private consultants. Some organisations have established offices in Cambodia (such as the Malaria Consortium and the Franco-Cambodian Pasteur Institut); others have conducted short-term projects. Prominent global health actors such as the Global Fund have also provided large grants to the health sector in Cambodia, further contributing to the generation of evidence, often in the form of reports and programme evaluations.

As a result of these developments, there has been a significant increase in health research output. However, some informants noted that the research landscape is fragmented, with little coordination between research projects sponsored by different organisations (CAM-01, 9 June 2014; CAM-09, 21 August 2014). Further, discrepancies were reported to exist between donor-funded research and health priorities in the country. As one high-level manager in the MoH remarked, “many times research is driven by funding, not demand. And this type of research is less relevant to the country” (CAM-05, 25 June 2014). Similarly, two other local informants, with many years of experience in the conduct and management of research programmes in Cambodia, complained that research and data collection efforts tend to focus on vertical programmes that receive donor support, but other important public health priorities for the country or wider health system analyses are neglected:

I believe hepatitis is a big problem in Cambodia; however, we have no data on this (…) What is the actual burden of hepatitis? No-one can answer this question, because we have no data. But we know everything on TB, HIV, and malaria (CAM-03, 17 June 2014).

The most active in research are the programmes like maternal and child health or HIV because there is external support and they are very specific. It is rare we have research from the perspective of the wider health system (CAM-02, 16 June 2014).

These perceptions are reflected in the results of a recent literature review, which found an increase in research output on communicable diseases (particularly malaria, HIV and tuberculosis), but under-representation of other important health issues in the country such as non-communicable diseases (which accounted for an estimated 34.5% of disease burden, but were the object of only 7.7% of publications in the period 2000–2012) as well as implementation and health system research.^33^ The same review also found that less than one-third of publications were led by an institution based in Cambodia.

### Institutional arrangements and the policy process

In addition to the need for both health data and research in Cambodia, it is important to consider the systems through which pieces of evidence can inform decisions. Over the past 15 years, following the reform of the health sector and the MoH, a number of institutional arrangements have been put in place, which appear well situated to provide evidence to key decision-making points. One notable example is the integration of the management of the NHIS and technical responsibility for strategic planning into a single structure of the MoH, the Department of Planning and Health Information. Specialised centres under the MoH also have organisational structures that may be conducive to the use of evidence. For example, the technical bureau of the National Centre for HIV/AIDS, Dermatology, and Sexually Transmitted Diseases incorporates a data management unit, a research unit and a planning unit, which are mandated to interact at various stages of data collection, reporting and planning (www.nchads.org).

The process of strategic development of the health sector has also been reformed and rationalised in ways that may provide various entry points for the use of evidence. These include, for example, the drafting of the multiannual Health Strategic Plan, the mid-term review of the Health Strategic Plan and annual health sector assessments such as the Joint Annual Performance Review and the Joint Annual Operational Plan appraisal. All these exercises are supported by various consultation mechanisms in which health data and research are routinely presented. Most notably, the Technical Working Group for Health (TWG-H) is a forum for policy dialogue and information sharing across a wide range of stakeholders, which was established in 2004 by the government of Cambodia to improve aid effectiveness, harmonisation and alignment with development partners. The TWG-H has a broad and inclusive membership, with subnational and civil society representation, and is based on monthly meetings, co-chaired by the Minister of Health (or a Secretary of State) and the WHO Country Representative. Directors of Provincial Health Departments (PHDs) are regularly invited to attend the meetings, where they usually provide a presentation on health progress and challenges in their administrations.

There was some consensus among participants that these mechanisms have created a well-functioning space for debate and coordination, contributing to the circulation of health information and knowledge among a wide range of stakeholders:

It is good to have forums such as the TWG to avoid duplication of efforts and find synergies between partners. Also, those meetings are crucial to promote an evidence-based culture because people meet and when they discuss they must support their arguments in a rational way, presenting evidence (CAM-03, 17 June 2014).

Participation of representatives from grassroots organisations and managers of provincial departments was mentioned as an important feature of the TWG-H, with the potential to enhance the visibility of local perspectives at the highest level of policy-making. In addition, the existence of consultative mechanisms at the village level might further support the generation and use of what has been termed "community-based evidence".^34^ Since the early 2000s, Village Health Support Groups have been established throughout the country to promote community participation, allowing elected community representatives to voice their concerns and needs at local meetings for the management of health facilities.^35^ In principle, this information can be disseminated both at the provincial and central level through the participation of local authorities in provincial working groups for health and the participation of directors of PHDs in the main TWG-H. As one informant noted, this is of great importance in Cambodia, as "you need to talk to people at the community level, as they know best what the problems are" (CAM-02, 16 June 2014).

Despite the existence of enabling structures, our investigation found gaps in the local context that make direct or widespread applications of evidence in line with best-practice expectations less likely. As informants pointed out, there are no clear guidelines about the way in which evidence should be appraised and used in policy processes. As a result, evidential practices were reported to be highly variable across different sectors and health issues, depending on the initiative and skills of individual managers and political will (CAM-03, 17 June 2014). High-ranking bureaucrats or politicians may require technical departments of the MoH or international organisations to provide evidence in support of policy-making and parliamentary debates. Yet, the lack of clear procedures, combined with power imbalances and the pressure of hierarchies, may constrain the ability of technical officers to act on, or even communicate, policy-relevant knowledge and information; one manager in the MoH explained, “we present evidence, but if a politician says, ‘I don’t believe it’, we cannot argue (…) we can present new evidence or clarify only if they request us to do so” (CAM-10, 27 August 2014). Further, mechanisms such as the TWG-H may serve well as a platform to share data and expertise. However, some informants noted that meetings tend to be very formal, especially when high-ranking politicians are present, and therefore their value as a forum to appraise and discuss evidence critically is limited (CAM-12, 25 August 2014, CAM-13, 25 August 2014). Similarly, an evaluation of the TWG-H found that meetings could be informed by "more substantive, strategic and open debate on policy, strategy and problem-solving, underpinned with evidence" (Wilkinson, p19).^36^

The use of evidence to inform decisions that require multisectoral coordination was seen as particularly problematic, especially for health policy decisions which have significant implications for the national budget, impinge on different agendas and require agreement across the political board. Interministerial committees have been established to promote dialogue on complex health policy issues such as tobacco control and nutrition, but the idea that health evidence alone can guide decision-making in these fora was questioned:

Anything inter-sectoral, like nutrition is very hard to get policy shift on. Because the Ministry of Agriculture says “well, look, we do what we can but our priority is food security” (…) And then you have to convince the Ministry of Health with technical evidence but, more importantly, it’s the business case to the Ministry of Economy and Finance, unless it’s a revenue neutral decision, but very few of them are (CAM-09, 21 August 2014).

The case of tobacco is a good illustration of these challenges. In April 2015, the Cambodian National Assembly approved the first ever Tobacco Law in the country, which introduced new restrictions on the import and sales of tobacco, smoking in public places and ban on most advertising. The approval of the Tobacco Law was a major step towards the implementation of the Framework Convention for Tobacco Control in Cambodia. However, the legislative process was very slow and unwieldy, destabilised by conflicting mandates of the MoH (to protect the health of the Cambodian population), the Ministry of Economy and Finance (to protect the national budget and therefore revenues generated by the sale of cigarettes) and the Ministry of Agriculture, Forestry, and Fisheries (to protect the agricultural sector and Cambodian tobacco farmers), in addition to pressures of tobacco corporations (CAM-08, 19 August 2014).

In the process, the advocacy of the National Center for Health Promotion of the MoH, the WHO and two NGOs was crucial to keep the issue on the agenda of the government and mobilise resources, including the production of evidence in the form of qualitative studies and surveys.^37–40^ Yet, the presentation of evidence about tobacco-related harms and high consumption rates in Cambodia was not sufficient to reach consensus, given the inconsistent mandates of different ministries. As one informant noted, “the Minister of Finance has a mandate to get more money, otherwise they have a big problem (…) so we had to give them evidence that increasing taxes is not a loss of revenue” (CAM-06, 27 June 2014). The same informant further explained:

Sometimes you need to present the same evidence in a different way (…), also because policy is multi-sectoral. It’s not that one minister decides. For example, they say that if you want to increase [tobacco] tax, this is not an issue of the Ministry of Health. We don’t have the power to do this. We can do a smoke-free policy, but tax is under the Ministry of Finance. So, you have to work closely with the Ministry of Finance… invite them to international workshops (…) Also, we have to explain that farmers do not rely on one crop only, so reduced tobacco production will not significantly affect them (CAM-06, 27 June 2014).

And when the Tobacco Law was approved in 2015, 12 years after the presentation of the first draft bill, the Prime Minister Hun Sen reportedly commented: “an individual cancer patient costs the government $10 000 per year, and the cost of treatment to the country is significantly higher than the $100 million spent by Cambodians on tobacco products” (The Phnom Penh Post, 9 April 2015). This kind of reasoning suggests that, at the highest level of decision-making, arguments about the economic impact of tobacco consumption were crucial to advancing the legislative process. This may not be surprising in a developing country that has placed economic growth at the centre of the development agenda and illustrates the limitations of oversimplified assumptions that health-related evidence not only speaks for itself but also will have an obvious political priority.

## Discussion

This study aimed to provide a broad mapping of challenges to, and opportunities for, the promotion of evidence-informed health policy-making in Cambodia, an approach which has been explicitly endorsed by the MoH and the global community alike. Our findings document a number of institutional and structural developments which may be conducive to meaningful and effective use of evidence for health policy and planning in the country; these include improved health information systems, increasing availability of institutional surveys and research products, the existence of participatory policy mechanisms in which evidence can be presented to local and international stakeholders and channels for the circulation of evidence across different levels of the MoH. However, other trends and features seem to be more problematic, including gaps between research areas and public health priorities, the fragmented nature of research activities and information systems, the lack of a national policy to support and guide the production and use of evidence for health policy, challenges to the use of evidence in support of intersectoral policy-making and the influence of external donors on research priorities. Some of these issues are known in health policy studies. For example, a recent literature review showed that development actors in many LMICs continue to operate research models that are not in line with widely accepted views of best practices, including a preference for vertical approaches to research capacity development, donor-led research agendas and fragmentary research programmes.^22^ Further, a recently published study in Cambodia and Pakistan found that stronger technical expertise (in terms of the ability to produce, interpret and disseminate knowledge) and the allocation of research funding to generate evidence in donor priority areas is an important means of donor influence on the policy process, leading to policies that are often not aligned with local priorities, needs and capacities.^19^ Yet, as noted in the same study, the implications of these power imbalances for the use of evidence in LMICs remain scarcely studied and warrant further consideration if we are to better understand prospects for the development of meaningful evidence advisory systems. In Cambodia, there have been good examples of knowledge translation where the development of context-specific solutions, informed by the generation of local evidence, has been a critical feature of policy and planning.^41–43^ However, our study highlighted remaining concerns about the lack of research and data-gathering activities to inform policy development in other important public health areas, such as non-communicable diseases, which have not received adequate support from external donors and the national government.

Further reflecting on our results, we can draw out general insights and recommendations, which can be useful to inform the process of resource allocation in the country as well as the wider debate in global health policy. First, as described, recent investments in Cambodia, as in many other LMICs, have tended to focus on knowledge generation, but little investments have comparatively been made to support local research organisations so that they can initiate, conduct and disseminate research findings independently. As emerged during the symposium "Building research capacity in Cambodia" (Phnom Penh, September 2015), a new generation of qualified researchers is ready to contribute to social development and innovation in the country, but more investments will no doubt be needed to provide them with the means to shape the research agenda. In particular, the strengthening of higher education institutions would be a key driver of innovation and development in the country, with potential spillover effects on the establishment of a culture of evidence. It would likely contribute to enhancing the status and recognition of the academic community in Cambodia, providing a critical mass of local experts. The development of research-oriented universities would also train a new generation of managers in public administration that are more familiar with research methods and practices, and thus in a better position to consider and evaluate evidence in policy processes. Efforts to achieve this will want to consider not just research training but also the development of a national research policy that increases the supply of local evidence and aligns research programmes with decision-making points and local needs to create the most favourable conditions for knowledge utilisation.

**Figure 1:**
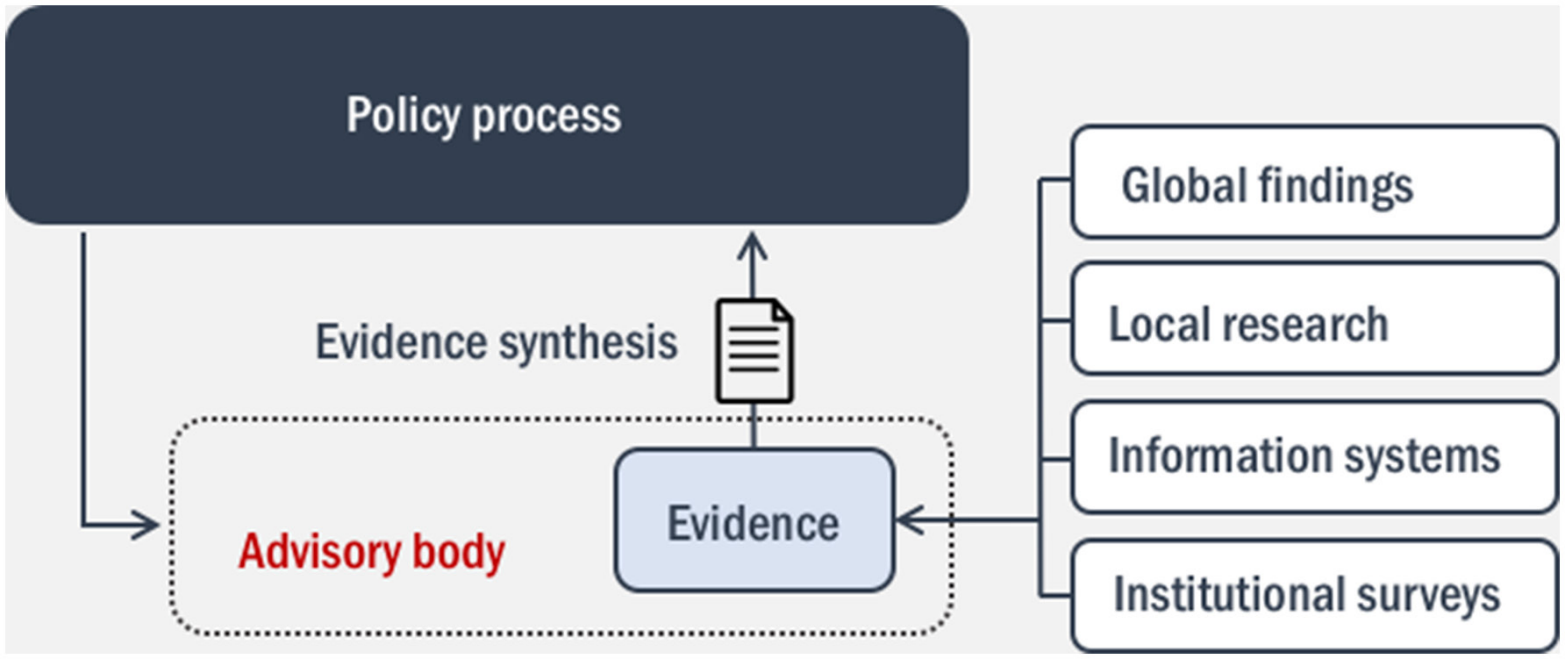
This diagram illustrates the function and value of an independent advisory body that can serve as central knowledge repository, review/integrate the diverse forms of evidence on particular health issues and produce evidence syntheses to inform the policy process.

Second, as we have seen, there are no clear guidelines and structures to support the use of evidence for health policy in Cambodia. Experiences in other LMICs indicate that dedicated committees, platforms and networks for knowledge management and translation can facilitate the regular and effective exchange of information between researchers and policy-makers^44–47^; however, as Hawkes *et al* pointed out a greater level of institutionalisation and regulatory support would enable stronger foundations for a continued and sustainable use of evidence.^48^ In Cambodia, the development of an independent advisory institute which can assess the quality of what is known on a particular issue, synthesise relevant data, information and knowledge, and provide decision-makers and key meetings of technical working groups with evidence-based policy briefs (or request research organisations to supply evidence on particular topics) would be an important step to address institutional gaps, especially to inform decisions that require multisectorial and multidisciplinary approaches or go beyond the simple adoption of global guidelines.^49^ This would be particularly useful given the proliferation of donor-backed quantitative and qualitative research output of variable quality (in addition to regional and global evidence), the fragmentation of routine information systems, and therefore the need for a specialised structure that can review, summarise, integrate and make the most out of available data and knowledge (figure 1). Further, a local group of advisers would be in a better position to navigate the complexity of informal social rules and power relations that are not discernible in official policy and regulations, but may influence pathways of knowledge translation in important ways. As one informant within the MoH noted, “the message is important in Cambodia, but the messenger is more important than the message (…) people trust other people, more than evidence” (CAM-10, 22 August 2014). In neighbouring Thailand, for example, the Health Systems Research Institute and the associated Health Intervention and Technology Assessment Program have provided effective institutional mechanisms to enable more explicit, rigorous and transparent policy-making.^50 51^ Local structures and mechanisms in Cambodia that can potentially exercise similar functions do exist. However, more resource should be allocated to support institutional development, and appropriate legislation should define mandate and responsibilities, while providing sufficient autonomy from domestic political pressures and donor interests. To this end, the advisory body could receive core funding from the national budget, but operate "at arm’s length" from the government—for example, through the appointment of the executive board by an independent commission, as seen in other countries.^52^

The development of enabling mechanisms and the strengthening of a culture of evidence will take time. Resources to support institutional development are still limited in Cambodia and partly dependent on international aid.^53^ In particular, the low salary levels at local universities do not provide an incentive to attract and retain the most qualified Cambodian researchers, who often studied or specialised abroad. There are, however, a number of indicators of structural arrangements that may increasingly work to serve an evidence advisory role in the health sector. In addition, if the country will continue to experience high rates of economic growth as in recent years,^54^ there may be increasing prospects for building local research capacities combined with institutional strengthening that can improve the relevance and utilisation of research products. Impact may be seen only in the long term, but it will be a major driver towards broader goals of self-reliance, local policy ownership and sustainable development.

Lastly, we should note some study limitations. Given the exploratory nature and broad scope of the research design, we could generate hypotheses and identify emerging issues and potential solutions, but further research is needed to verify or explore them in-depth. For example, (participant) observations at key meetings of technical working groups would provide a thicker description of dynamics and interactions between local and international stakeholders, and specific ways in which different types of evidence are presented, discussed and valued, including local data and surveys, findings from other countries and experiences from the communities. It would also be relevant to explore in detail such interactions in the conduct of research projects and the extent to which the involvement of local researchers can facilitate the generation of findings that are tailored to the local context and knowledge translation. Second, we could identify a number of institutional, political and structural features which may affect how and what evidence is used for health policy and planning. However, a detailed examination of the complexity of competing actors and interests influencing policy directions (in ways that are not necessarily informed by evidence) was beyond the aim and scope of this study and would require the in-depth analysis of specific case studies. Finally, when investigating policy issues and the views of government officers in particular, a tendency to provide socially desirable accounts, disengaged from controversial issues, can potentially result in biased accounts. In our interviews, we tried to minimise the potential for such bias by prioritising "how" questions rather than "why" questions, as these are known to create a more defensive attitude.^55^ Further, we did not find major contradictions and discrepancies between the accounts of different categories of informants; however, higher level local managers tended to provide more formal views, reflecting official government statements, while mid-level cadres and international stakeholders tended to give more critical and elaborated accounts.

## Conclusions

This study highlights a number of developments in Cambodia that may increasingly work to facilitate the supply of health-related data and information, and their use to inform policy and planning. It also identified ways in which the institutional framework could be strengthened to create more favourable conditions to support evidence-informed policy-making. The issues we have discussed here are complex, intertwined and shaped in multidimensional ways by contextual factors. Yet, lessons from Cambodia can be useful to explore and better understand the environment for evidence-informed policy in many other contexts, especially in transitional economies where national structures for the generation and use of policy-relevant evidence are not yet fully developed and where research priorities have been directed by external actors.^56^ In such contexts, further studies are needed to capture key contextual and institutional variables and their influence on evidence-informed advisory systems. In this paper, we have offered a set of concepts and insights to explore these issues, which can hopefully be refined and used to inform research design for other investigations.
